# Correlation of Balloon Pressure Used for Pneumatic Dilatation in Achalasia with Manometric Findings and Factors Associated With the Need for Repeat Procedure

**DOI:** 10.7759/cureus.65623

**Published:** 2024-07-29

**Authors:** Muhammad Furrukh, Tayyab S Akhter, Fawad Rahman, Fatima Ayaz, Saima Ambreen

**Affiliations:** 1 Department of Medicine, Holy Family Hospital, Rawalpindi, PAK; 2 Department of Gastroenterology and Hepatology, Holy Family Hospital, Rawalpindi, PAK; 3 Department of Medicine, Combined Military Hospital, Rawalpindi, PAK

**Keywords:** high resolution manometry, high-resolution esophageal manometry, balloon dilatation, pneumatic dilation for achalasia, esophageal achalasia

## Abstract

Achalasia is a chronic and rare disorder of esophageal motility. It is characterized by spastic or absent esophageal contractions and impairment of relaxation of the lower esophageal sphincter. Treatment modalities include smooth muscle-relaxing medications, botulinum toxin injections to lower the esophageal sphincter, pneumatic dilatation, and surgical interventions. Pneumatic dilatation is deemed to be an effective treatment option and is the most widely used non-surgical intervention. We designed this prospective study to look for any possible correlation between balloon pressure used in pneumatic dilatation, manometric findings, and patient characteristics. And to find any possible association between the need for repeat pneumatic dilatations and factors like gender, age, duration of symptoms, Eckardt score, daily retrosternal pain, manometric findings, and balloon pressures. Thirty-one patients with confirmed achalasia were enrolled in the study. All of these patients underwent pneumatic dilatation. Pearson's correlation coefficient was found to be 0.234 (p-value 0.23) between the required balloon pressure and integrated relaxation pressure (IRP). Six of these patients required repeat pneumatic dilatations. No statistically significant association was noted between the need for repeat intervention and gender, age over 40, age under 20, Eckardt score over 10, daily chest pain, duration of symptoms over two years, and IRP over 30 mmHg. In conclusion, it could be said that pneumatic dilatation does not carry a 100% success rate, and repeat sessions are needed in many of the patients with achalasia. So, long-term follow-up is crucial. Managing expectations and making a realistic plan with proper informational care is important at the beginning of treatment.

## Introduction

Achalasia is a chronic and rare disorder of esophageal motility. It is characterized by spastic or absent esophageal contractions and impairment of relaxation of the lower esophageal sphincter (LES) [1,2]. The underlying mechanism is not fully understood. Loss of inhibitory nerve fibers in the esophageal myenteric plexus is believed to be the key underlying pathology. Various genetic and environmental factors are implicated in the pathogenesis. These factors lead to a series of inflammatory and immune responses, which ultimately destroy inhibitory neurons in the myenteric plexus [1,3]. The incidence of achalasia increases with advancing age, but it can affect all age groups and has no significant predilection to any gender or race [4,5]. It can present with a multitude of symptoms, including dysphagia, heartburn, food regurgitation, chest pain, weight loss, and a deficiency of nutrients [4-6]. Various diagnostic investigations, like high-resolution manometry, endoscopy, and barium esophagograms, are available. Achalasia is not fully curable, but adequate treatment can ensure near-normal swallowing and symptom control. Treatment modalities include smooth muscle-relaxing medications, botulinum toxin injections for LES, pneumatic dilatation, and surgical interventions [6-8].

Pneumatic dilatation is deemed to be an effective treatment option. It is the most widely used non-surgical intervention. The pneumatic dilators are commonly available in three sizes: 30mm, 35mm, and 40mm. Using a larger-diameter dilator initially increases the likelihood of success but carries a higher risk of esophageal perforation. So, these dilators are usually used in a graded fashion (30 mm first, followed by 35 mm, and then 40 mm, if required). Treatment success rates vary from 50 to 93% in various studies [9-11]. Many factors have been identified that correlate with poor outcomes. Studies have shown that younger age, male gender, improper balloon positioning, and high post-therapy lower esophageal sphincter pressure are linked to lower immediate success rates and poor long-term results [8-13]. Unfortunately, local data is particularly lacking in terms of predictors of the outcome of pneumatic dilatation. We designed this prospective study to look for any possible correlation between balloon pressure used in pneumatic dilatation, manometric findings, and patient characteristics. And to find any possible association between the need for repeat pneumatic dilatations and factors like gender, age, duration of symptoms, Eckardt score, daily retrosternal pain, manometric findings, and balloon pressures. 

## Materials and methods

This prospective study was carried out at the Department of Gastroenterology, Holy Family Hospital, Rawalpindi, Pakistan. The project was approved by the research and ethical committee of the institution (reference number: R-59/RMU). A total of 31 patients were enrolled in the study via consecutive non-probability sampling over the course of six months (December 2019 to June 2020). Patients underwent pneumatic dilatation after informed consent and were followed up for three years for the development of recurrent symptoms.

Inclusion criteria

All patients with a confirmed diagnosis of achalasia were included in the study.

Exclusion criteria

Severe cardio-respiratory diseases, severe coagulopathy, and pharyngeal or esophageal malignancies were set as exclusion criteria.

Operational definitions

Achalasia

Confirmed achalasia was defined as clinically suspected achalasia with an Eckardt score of 3 or more, with or without positive manometric findings.

Integrated Relaxation Pressure

Integrated relaxation pressure (IRP) was defined as the average lowest pressure through the esophagogastric junction for 4 seconds (either contiguous or non-contiguous) within the 10-second window following deglutitive upper esophageal sphincter relaxation. Three of the patients could not undergo manometry (two due to severe esophageal tightness and the third due to non-cooperation). 

Pneumatic Dilatation

Endoscopy was performed under conscious sedation, and a Rigiflex balloon was passed over the guidewire, which was positioned across the gastro-esophageal junction and inflated to a pressure of 5-15 PSI (until obliteration of the waist) for at least 60 seconds. Initially, a 30 mm balloon was used in all the patients. In the event of failure to improve symptoms to a functional status (Eckardt score 2 or less), the procedure was repeated with a 35mm after at least 4-6 weeks. It was further followed by a 40-mm balloon in case of persistence of symptoms (Eckardt score of 3 or more).

Eckardt score

The Eckardt score is frequently employed for the initial evaluation of symptoms and to gauge the efficacy of achalasia treatment [13]. Dysphagia, regurgitation, chest pain, and weight loss are included in the scoring, and every symptom is attributed up to three points. It is further elaborated in Table 1. 

**Table 1:** Eckardt score for symptomatic evaluation in achalasia [13]

Score	Weight loss	Dysphagia	Retrosternal pain pain	Regurgitation
0	None	None	None	None
1	<5 kg	Occasional	Occasional	Occasional
2	5-10 kg	Daily	Daily	Daily
3	>10 kg	Each meal	Each meal	Each meal

Statistical analysis

All the collected data was entered in IBM Corp. Released 2017. IBM SPSS Statistics for Windows, Version 25.0. Armonk, NY: IBM Corp. Means were calculated for all the continuous variables, whereas frequencies were calculated for categorical variables. The null hypothesis was formulated that there is no association between the need for repeat dilatation after the first pneumatic dilatation and age under 20 or over 40 years, gender, Eckardt score of more than 10, IRP of over 30 mmHg, duration of symptoms over two years, daily chest pain, and use of balloon pressure over 10 PSI. Fisher's exact test was applied to test the hypothesis, and a p-value of less than 0.05 was deemed statistically significant. Furthermore, the Pearson correlation coefficient was calculated to look for any possible correlation between balloon pressure and integrated relaxation pressure (IRP), Eckardt score, duration of symptoms, and age of the patients. 

## Results

Out of 31 patients, 17 (54.8%) were male and 14 (45.2%) were female. The average age of the participants was 36.71 (±14.92) years. The average duration of symptoms was 27.1 (±18.05) months. The study population had an average Eckardt score of 8.45 (±2.3). Integrated relaxation pressure (IRP) ranged from 13.79 mmHg to 95.33 mmHg, with a mean value of 30.58 mmHg (±15.23). The average balloon pressure required for pneumatic dilatation was found to be 8.48 PSI (±2.30). Pearson's correlation coefficient was calculated for required balloon pressure with age, duration of symptoms, Eckardt score at presentation, and IRP, as shown in Table 2.

**Table 2:** Correlation of balloon pressure with various factors

Parameter	Pearson’s co-efficient	p-value
IRP	0.234	0.230
Age	0.115	0.539
Duration of symptoms	0.303	0.097
Eckardt score	-0.034	0.855

After the first pneumatic dilatation with a balloon size of 30 mm, 25 patients did not require further treatment over the course of three years. The rest of the six patients underwent a second pneumatic dilatation with a balloon size of 35 mm. Fischer’s exact test was applied to look for any association between the use of balloon pressure over 10 PSI and the need for repeat treatment. A p-value of 0.596 was obtained, which confirmed the null hypothesis that the use of balloon pressure over 10 PSI had no significant association with the need for retreatment. The Fischer exact test was also applied to look for any possible association of gender, age over 40 years or under 20 years, Eckardt score over 10, daily chest pain, duration of symptoms over two years, and IRP over 30 mmHg with the need for a repeat procedure. For all these variables, no significant association was found, and the p-values were noted to be 0.185, 0.67, 1.0, 0.55, 1.0, 0.638, and 1.0, respectively, confirming the null hypothesis.

## Discussion

Achalasia is known to be a chronic disorder and requires long-term management and follow-up. Without treatment, achalasia can lead to progressive dilation of the esophagus, Barrett’s esophagus, and esophageal carcinoma [2]. The main aim of the management of achalasia is symptom control and prevention of complications by improving esophageal emptying. Options for definitive management include pneumatic dilation, per-oral endoscopic myotomy, and myotomy. Other nondefinitive therapies, like medications and botulinum toxin, could be tried for symptomatic relief. Esophagectomy is usually reserved for those patients who have failed to respond to other therapies [2,5]. A variety of factors influence the choice of therapy, such as the overall state of the patient's health, patient preference, and the availability of resources. Pneumatic dilatation (PD) is the most effective and common non-surgical treatment option for achalasia [11-15].

The mean age of the patients in our study was found to be 36.71 (±14.92) years. Whereas the studies conducted by Farhoomand, Alderstein, and Boeckxstaens showed the average age of participants to be 45.8, 50, and 46.4 years, respectively [11,12,16]. In our study, the male-to-female ratio was 1.21, while studies conducted by Farhoomand, Eckardt, and Alderliesten showed the ratio to be 1.85, 1.57, and 0.92, respectively [11-13]. The average Eckardt score in our study population was 8.45 (±2.3), whereas studies conducted by Eckardt and Gupta showed a mean Eckardt score of 7.9 (±2.3) and 7.16 (±0.834), respectively [13,14].

Various studies have reported success rates of pneumatic dilatation ranging from 15% to 85%. This variability is potentially due to the use of different study definitions, periods of follow-up, and treatment regimens. The results of our study fit within the above-mentioned range of success. However, there were no studies conducted specifically to look for any possible correlation between balloon pressure and manometric findings, patient characteristics, or the need for repeat procedures. Ponce and colleagues found out that age younger than 20 years and male gender were associated with poor outcomes [15]. Alderstein and colleagues noted that age younger than 40 was associated with the need for repeat intervention, while gender had no association with it [12]. Boeckxstaens and colleagues found daily chest pain and age under 40 years were associated with the need for repeat dilatation, while gender had no significant association with the need for repeat dilatation [16]. In contrast to them, we found no relation between age, gender, or chest pain and the need for a repeat procedure.

Limitations of our study include a small sample size, non-probability sampling, single-center-based experience, and relatively short follow-up. Further, large multi-centered studies with longer follow-ups can be organized to investigate the relation and impact of these studied factors on the recurrence of symptoms and the need for repeat intervention.

## Conclusions

Pneumatic dilatation does not carry a 100% success rate, and often repeat sessions are needed. Therefore, a long-term follow-up is crucial. It can be concluded that the success of initial pneumatic dilatation is not significantly affected by age, gender, duration of symptoms, initial Eckardt score, or manometric findings. Managing expectations and making a realistic plan with proper informational care is important at the start of treatment.
